# Gender-specific impacts of thigh skinfold thickness and grip strength for predicting osteoporosis in type 2 diabetes

**DOI:** 10.1186/s13098-023-01087-w

**Published:** 2023-05-18

**Authors:** Chieh-Hua Lu, Chien-Hsing Lee, Li-Wei Wu, Chun-Cheng Liao, Sheng-Chiang Su, Jhih-Syuan Liu, Peng-Fei Li, Chia-Luen Huang, Li-Ju Ho, Chien-Ming Lin, Ming-Hsun Lin, Chun-Yung Chang, Yi-Chen Liu, Chih-Ping Lin, An-Che Cheng, Feng-Chih Kuo

**Affiliations:** 1 Division of Endocrinology and Metabolism, Department of Internal Medicine, Tri-Service General Hospital, National Defense Medical Center, 325, Section 2, Cheng-Kung Road, 114, Taipei, Taiwan, ROC; 2 Division of Family Medicine, Department of Family and Community Medicine, Tri-Service General Hospital, School of Medicine, National Defense Medical Center, Taipei, Taiwan, ROC; 3 Division of Geriatric Medicine, Department of Family and Community Medicine, Tri-Service General Hospital, School of Medicine, National Defense Medical Center, Taipei, Taiwan, ROC; 4 Graduate Institute of Medical Sciences, National Defense Medical Center, Taipei, Taiwan, ROC; 5 Department of Family Medicine, Taichung Armed Forces General Hospital, Taichung, Taiwan, ROC; 6 School of Medicine, National Defense Medical Center, Taipei, Taiwan, ROC; 7 Department of Medical Education and Research, Taichung Armed Forces General Hospital, Taichung, Taiwan, ROC; 8 Department of Pediatrics, Tri-Service General Hospital, National Defense Medical Center, Taipei, Taiwan, ROC; 9 Division of Endocrinology and Metabolism, Department of Internal Medicine, Kaohsiung Armed Forces General Hospital, Kaohsiung, Taiwan, ROC

**Keywords:** Type 2 diabetes, Grip strength, Skinfold measurement, Dual-energy x-ray absorptiometry, Osteoporosis, Body composition

## Abstract

**Background:**

Diabetes with co-existing bone fragility or osteoporosis is common in elderly patients, whereas is frequently underestimated.

**Methods:**

We conducted dual-energy x-ray absorptiometry (DXA) with 7-site skinfold (SF) and dominant hand grip strength measurements among patients with type 2 diabetes (T2DM) to assess their gender-specific associations. A total of 103 patients with T2DM (60 females and 43 males), aged between 50 and 80 years (median 68.0 years) were enrolled and 45 non-DM females were also included to compare with T2DM females.

**Results:**

Our results revealed osteoporosis was negatively correlated with grip strength in both genders, negatively correlated with lean mass solely in males and negatively correlated with fat mass (particular the gynoid fat mass and thigh SF thickness) in females. Via performing multivariable stepwise logistic regression, we identified grip strength in both genders and thigh SF thickness in females as predictors for osteoporosis. Receiver operating characteristic curve analysis further disclosed 20.5 mm female thigh skinfold thickness, 18.1 kg female grip strength and 29.0 kg male grip strength as reasonable cutoff levels for predicting osteoporosis in the Taiwanese patients with T2DM.

**Conclusions:**

Patients with T2DM presented gender-specific associations between osteoporosis, body composition and grip strength. Grip strength and thigh SF thickness might serve as predictors for detection of osteoporosis in patients with T2DM.

**Supplementary Information:**

The online version contains supplementary material available at 10.1186/s13098-023-01087-w.

## Introduction

The prevalence of type 2 diabetes rises rapidly worldwide, and the Asia-Pacific region is at the forefront of the diabetes epidemics with around 138 million people (8–9% prevalence) were living with diabetes in 2013, and the number is expected to increase to 201.8 million by 2035 [1]. Based on the Taiwan National Health Insurance Research database in 2014, the overall prevalence of diabetes in Taiwan is around 9.4% and more than half (50.3%) are aged ≥ 65 years. It is worth noting that the prevalence of diabetes in those aged ≥ 65 years can reach approximately 40% [2]. Meanwhile, the risk of osteoporosis also increases with aging [3]. Hence, the co-existence of diabetes and osteoporosis will be remarkably raised in the aged people. This issue will be especially crucial in East Asia region since the burden of osteoporosis and its associated fragility fractures are expected to increase substantially [4]. Considering osteoporotic fractures will lead to drastic increase of morbidity, mortality, and medical cost [5] and patients with diabetes are more vulnerable to falling down [6], identification of the patients with diabetes who have concurrent osteoporosis will be vital and is still largely under diagnosed in the current clinical practice.

The gold standard for diagnosis of osteoporosis is based on the bone marrow density (BMD) measured via dual-energy x-ray absorptiometry (DXA) scans of hip or spine in individuals over aged 50 years. A T-score of -2.5 or lower indicates the presence of osteoporosis [7]. Notably, recent report has disclosed that patients with type 2 diabetes (T2DM) have increased risk for osteoporosis in the younger age [8] and International Osteoporosis Foundation (IOF) have advised to screen BMD in all patients with diabetes aged older than 50 [9]. However, it is difficult to perform universal BMD measurement in all patients with diabetes above 50 years old since DXA is not generally accessible in the outpatient clinic. Besides, the fracture risk assessment tool (FRAX) as the algorism to evaluate the 10-year probability of fracture (https://www.shef.ac.uk/FRAX/tool.jsp) did not include type 2 diabetes as one of the risk factors and has been shown to underestimate the fracture risk in patients with diabetes [10]. Therefore, there is still an unmet need to employ a practical tool for detecting the patients with diabetes who are likely to have concurrent osteoporosis.

Recent studies have revealed the occurrence of osteoporosis is likely associated with changes of several human body components or muscular function. For example, lean mass and grip strength has been found to be positively associated with BMD in physically active postmenopausal women [11]. Fat mass loss was associated with lumber spine bone loss in women aged 50 years or older [12]. Also, a low skinfold thickness appears to be an indicator of osteoporosis in a group of osteoporotic women and normal controls [13]. However, there is still very limited investigation to evaluate the inter-associations of osteoporosis with different human body components and muscular function in patients with diabetes. Particularly, there is very rare research focusing on the gender-specific differences on their inter-associations.

Therefore, in this study, we recruited patients with T2DM aged 50–80 years old to receive DXA scanning as a gold standard for diagnosis of osteoporosis [7] and a standard method for precisely quantifying regional human body components [14, 15]. We also concurrently utilized skinfold caliper [16] and grip electronic dynameter [17] to measure 7-site skinfold thickness and dominant hand grip strength, respectively. A group of 45 non-DM females, aged 23–72 years old was also included to compare with T2DM females. We hypothesized these detailed anthropomorphic measurements will reveal the gender specific inter-associations of osteoporosis with multiple human body components (regional bone mineral density, lean mass, fat mass and 7-site skinfold thickness) and grip strength and will provide valuable information for detection of patients with T2DM who are at high risk of being osteoporotic. Besides, measurements of skinfold thickness and grip strength could be easily conducted in the outpatient department and might serve as a practical tool. Therefore, we particularly focus on their potential roles for predicting osteoporosis in patients with T2DM under regular outpatient follow-up.

## Materials and methods

### Study population and sample size estimation

Total, 103 patients with T2DM (60 females and 43 males) and 45 non-DM females were recruited from outpatient department of Endocrinology and Metabolism, Tri-Service General Hospital during regular follow-up. The criteria for inclusion into this trial were as follows: patients with T2DM age between 50 and 80 years with stable condition under regular treatment with either oral hypoglycemic agents or injection therapy of insulin or glucagon-like peptide 1 receptor agonist. For comparing to T2DM females, non-DM females age between 20 and 80 years were further included for analysis. Individuals with pregnancy, current acute illness of cerebrovascular accident, myocardial infarction, heart failure, renal failure, hepatic failure, or psychiatric diseases were excluded. There are no participants who received chronic treatment with sex hormones or glucocorticoids. Written informed consent was signed before participating in this study and the use of relevant personal information was agreed on a confidential basis. The institutional review boards of Tri-Service General Hospital (TSGH) approved this study. (TSGHIRB number: 2-108-05-052)

For estimating the required sample size, we applied MedCalc® Statistical Software version 20.305 (MedCalc Software Ltd, Ostend, Belgium) with selection of type I error as α = 0.05 and type II error as β = 0.2 (power: 80%). According to previous reports, skinfold thickness and grip strength were significantly correlated with bone mineral density (r = 0.486 and 0.482) [18, 19] and could serve as predictors for osteoporotic fractures or falls with AUC of 0.66 and 0.71 [20, 21], respectively. Therefore, the minimum required sample size for correlation analysis will be n = 31 via inputting the anticipated correlation coefficient with either 0.486 or 0.482. Also, provided we expected to show the AUC of 0.735 in female skinfold thickness and AUC of 0.850 in male grip strength and applied the ratio of non-osteoporotic/osteoporotic subjects in our data (2.5 in T2DM females and 5.1 in T2DM males), the minimum required sample size for ROC curve analysis with be n = 56 in females and n = 37 in males.

### Anthropometric measurements

Measurements of body weight and standing height were performed using a standard scale and a wall-mounted stadiometer, respectively as barefoot with the patients wearing light indoor clothing. Body weight was recorded to the nearest 0.1 kg; body height was recorded to the nearest 0.1 cm. Measurement of waist circumference was performed at the midway horizontal plane between the inferior margin of the last rib and the crest of the ilium. Measurement of hip circumference was performed at its widest point. Waist circumference and hip circumference were recorded to the nearest 0.1 cm. Waist-to-hip circumference ratio and body mass index (BMI) were calculated. BMI was calculated as weight in kilograms divided by the square of height in meters. Blood pressure measurement from the right arm was performed in a sitting position after resting for 5 min and repeated measurement was done one minute later. The average value of blood pressure was used in the analysis.

### Hand grip strength measurement

Hand grip strength was measured using an electronic hand dynamometer. The width of dynamometer was adjusted for optimal fit for each participant. Measurement was performed on the dominant hand and recorded to the nearest 0.1 kg. All participants were instructed to handle the dynamometer beside without against their body and keep the elbow bent at a 90-degree angle, then squeeze the dynamometer with maximal effort. Tests were performed twice and the greatest one was used in the analysis.

### Skinfold thickness measurement

The standard protocols of skinfold measurement are as recommended by the Committee on Nutritional Anthropometry of the Food and Nutrition Board of the National Research Council [22]. Measurements of skinfold thicknesses were performed using Lange skinfold caliper and conducted by a single experienced technician to avoid the variation between observers. All the skinfold thicknesses were measured twice with the differences of two readings less than 2 mm and recorded to the nearest 0.1 mm. The average values were used for analysis. In each individual, total 7-different sites of the right-side body were measured including tricep, subscapular, chest, midaxillary, suprailiac, abdominal and thigh skinfold thicknesses. The android skinfold thickness was further calculated as the sum of suprailiac and abdominal skinfold thickness. The thigh-to-android skinfold ratio was used as the surrogate for peripheral fat distribution.

### Dual-energy X-ray absorptiometry

Dual-energy X-ray absorptiometry (DXA) was performed following the guidelines of International Society for Clinical Densitometry [23] to quantify BMD and T-score over lumbar spine (L1-L4) and bilateral femoral neck regions. In accordance with the recommendation of the Committee of Scientific Advisors of International Osteoporosis Foundation (IOF), Caucasian women aged 20–29 years from the National Health and Nutrition Examination Survey (NHANES) database were used as the reference for T-score calculation [24]. Any one of spine or femoral neck regions with a T-score of -2.5 or lower indicates the presence of osteoporosis and T-score between − 1 to -2.5 was defined as osteopenia [7]. DXA was also applied as a standard method to quantify whole and regional body components, including fat mass, lean mass, and bone mineral content. DXA was operated by a certificated technician and participants were dressed in cotton robes without metal attachments, lying in a supine position in the center of the scanning field with their palms facing downwards, arms positioning away from their body, and feet, face, chin all maintaining in the neutral position. The type of the DXA machine is Lunar Prodigy Advance enCORE 2011 equipped with the DXA software (enCORE V13.60.033).

### Biochemical variables measurement

Venus blood samples were drawn under an 8-hour fast status. Biochemical data including glucose, lipids, liver, and renal function were measured. Serum levels of triglycerides, low-density lipoprotein (LDL) cholesterol, alanine aminotransferase (ALT) and creatinine were assessed by a Beckman Synchron LX20 analyzer (LX20; Beckman Coulter, Brea, CA, USA). Plasma glucose concentrations were determined on a Beckman Glucose Analyzer II (Beckman Instruments, Fullerton, CA, USA) with the glucose oxidase method. Calibration and quality control with standard solutions were performed ahead before measuring the biochemical data. Repeat measurement will be performed if abnormal data were observed. Biochemistry data were collected from recent 3-months medical records away from the date of DXA, anthropometric, grip strength and skinfold measurements.

### Statistical analysis

Continuous variables were presented as median values with quartiles and analyzed by the Mann-Whiney U-test. Categorical variables were presented as percentages and assessed using Chi-square test. Statistical significance was defined as p value less than 0.05. Correlations of osteoporosis with various variables were analyzed using Spearman rank-order correlations in gender-specific manner. Multivariable stepwise logistic regression was applied to evaluate the gender-specific predictors for osteoporosis. Receiver Operating Characteristic (ROC) curve was further drawn to identify the gender-specific cutoff levels of thigh skinfold or grip strength for predicting osteoporosis. All statistical analyses were performed using SPSS software version 22 (IBM, Chicago, Illinois, USA).

## Results

### Characteristics of the study population

Comparisons of basic anthropometric characteristics, biochemical data, and medical prescription between two genders of patients with T2DM were shown in Table 1. In general, females present significantly lower waist-to-hip ratio, lower serum creatinine levels and lower prevalence of smoking and alcohol drinking than males. Both genders are in the similar range of age (median 68.8 years in females and 67.5 in males), BMI (median 25 kg/m^2^ in females and 26.1 kg/m^2^ in males) and duration of diabetes (median 10 years in both). Also, both groups are under stable disease status without significant differences on blood pressure, glycemic control, and lipid profiles. There are also no significant differences between genders on the prescriptions of statin, oral hypoglycemic agents, or injectable anti-diabetic regiments. Particularly, there is no gender-specific difference on the prescription of thiazolidinedione (TZD), which has been shown to increase the risk of osteoporosis [25] and alter the fat distribution [26]. Other anti-diabetic agents that could potentially change weight or body composition, such as sodium-glucose co-transporter 2 inhibitor (SGLT2i), insulin and glucagon-like peptide 1 receptor agonist (GLP1RA) were also prescribed in both groups without significant difference. We also compared the 60 T2DM with 45 non-DM females (age between 20 and 80 years) and 11 non-DM females (age between 50 and 80 years) as shown in Supplementary Table 1. The 45 non-DM females were younger with lower BMI, lower waist-to-hip ratio and significantly better conditions in blood pressure, triglyceride, creatinine, ALT, and glucose levels, but higher LDL levels, which is likely due to the use of statin in DM subjects. Whereas the 11 non-DM females were only around 3 years younger with similar status in BMI, waist-to-hip ratio, blood pressure, creatinine, ALT and lower glucose levels, but higher lipid profiles (LDL and triglyceride).

**Table 1:** Basic anthropometric characteristics, biochemical data, and medical prescription in the T2DM patients

	Females	Males	*p* value
	(n = 60)	(n = 43)	
Age (years)	68.8 [63.2; 72.6]	67.5 [60.3; 71.4]	0.164
BMI (kg/m^2^)	25.0 [21.9; 27.2]	26.1 [23.1; 29.8]	0.082
Waist-to-hip ratio	0.94 [0.89; 1.01]	0.98 [0.92; 1.04]	0.032*
Systolic BP (mmHg)	134 [123; 146]	134 [123; 145]	0.807
Diastolic BP (mmHg)	74 [67; 81]	79 [72; 83]	0.095
Fasting glucose (mg/dL)	129 [105; 153]	131 [112; 147]	0.896
HbA1c % HbA1c (mmol/mol)	7.5 [6.6; 8.2] (58 [49; 66])	7.1 [6.6; 8.1] (54 [49; 65])	0.388
LDL cholesterol (mg/dL)	91 [71; 105]	80 [69; 102]	0.360
Triglyceride (mg/dL)	108 [77; 136]	101 [76; 155]	0.960
Creatinine (mg/dL)	0.7 [0.6; 0.8]	1.0 [0.9; 1.2]	< 0.001**
ALT (U/L)	18 [15; 25]	20 [13; 26]	0.804
Years of diabetes	10 [6; 20]	10 [5; 19]	0.719
Smoking % (n)	0% (0)	30.2% (13)	< 0.001**
Alcohol drinking % (n)	1.7% (1)	32.6% (14)	< 0.001**
Statin % (n)	81.7% (49)	74.4% (32)	0.376
Metformin % (n)	73.3% (44)	83.7% (36)	0.212
AGI % (n)	8.3% (5)	14% (6)	0.362
SU or glinide % (n)	51.7% (31)	62.8% (27)	0.262
TZD % (n)	6.7% (4)	14% (6)	0.218
SGLT2i % (n)	26.7% (16)	44.2% (19)	0.064
DPP4i % (n)	33.3% (20)	27.9% (12)	0.557
GLP1RA % (n)	13.3% (8)	11.6% (5)	0.797
Insulin % (n)	35% (21)	27.9% (12)	0.447

Continuous variables were analyzed using the Mann-Whitney U-test and are presented as median values and [quartiles]; Categorical variables were analyzed using the Chi-square test and are presented as percentages (number). Abbreviations: BMI, body mass index; BP, blood pressure; HbA1c, glycated hemoglobin; LDL, low density lipoprotein; ALT, alanine aminotransferase; AGI, alpha glucosidase inhibitor; SU, sulfonylurea; TZD, thiazolidinedione; SGLT2i, sodium-glucose co-transporter 2 inhibitor; DPP4i, dipeptidyl peptidase 4 inhibitor;

GLP1RA, glucagon-like peptide 1 receptor agonist. *p < 0.05; **p < 0.001

### Comparison of grip strength and body composition between genders and DM/non-DM females

We further compared the gender differences among hand dynamometer assessed grip strength, DXA and skinfold caliper measured regional body composition/skinfold (SF) thickness and DXA defined osteopenia/osteoporosis in the patients with T2DM as listed in Table 2. Clearly, males have significantly higher grip strength, higher total and regional (arms, legs, android, gynoid) lean mass, and higher bone mass (total bone mineral content, lumbar BMD, and bilateral femoral neck BMD) than females. There is no significant gender difference on total fat mass, whereas females present predominant peripheral fat distribution (higher arms fat mass, higher tricep and thigh SF thickness, and higher thigh to android SF ratio) and males have significantly increased central fat accumulation (higher android to gynoid fat ratio). In turns of the prevalence of osteopenia and osteoporosis, there is no significant difference between genders, but females have a non-significant trend with higher prevalence of osteoporosis than males (28.3% versus 16.3%, *p* = 0.154). Besides, we also performed the same comparisons between 60 T2DM females, 45 non-DM females (age between 20 and 80 years) and 11 non-DM females (age between 50 and 80 years) as shown in Supplementary Table 2. The 45 non-DM females presented higher grip strength, similar total lean mass, but distinguished regional lean mass (higher in leg and lower in android), and higher bone mass (total bone mineral content, lumbar BMD, and bilateral femoral neck BMD). They also had lower total fat mass with better feminine body shape (lower android fat, higher gynoid fat with lower android to gynoid fat ratio and higher thigh to android SF ratio) and lower skinfold thickness in midaxillary, subscapular, abdominal and suprailicac regions with decreased prevalence of osteoporosis. Regarding to the 11 non-DM females, they presented similar body composition with the T2DM females, but better feminine body shape (lower android to gynoid fat ratio and higher thigh to android SF ratio) with higher grip strength and lower skinfold thickness in chest and subscapular regions.

**Table 2:** Gender difference among grip strength, DXA scan parameters, skinfold measurement and osteoporosis/osteopenia in the T2DM patients

	Females	Males	*p* value
	(n = 60)	(n = 43)	
**Grip strength (kg)**	17.9 [15.2; 21.7]	34.8 [27; 39.2]	< 0.001**
**DXA scan parameters**			
Total lean mass (kg)	34.7 [31.8; 38.3]	50.7 [46.6; 54.4]	< 0.001**
Total fat mass (kg)	20.5 [15.3; 25]	21 [12.6; 27.6]	0.831
Total BMC (kg)	1.92 [1.67; 2.21]	2.74 [2.41; 2.96]	< 0.001**
Arms lean mass (kg)	3.42 [3.07; 3.83]	5.52 [5.03; 6.04]	< 0.001**
Legs lean mass (kg)	10.4 [9.9; 11.2]	15.4 [14; 16.7]	< 0.001**
Android lean mass (kg)	2.47 [2.15; 2.76]	3.73 [3.32; 4.10]	< 0.001**
Gynoid lean mass (kg)	4.68 [4.19; 5.01]	6.99 [6.44; 7.59]	< 0.001**
Arms fat mass (kg)	2.11 [1.59; 2.78]	1.51 [1.12; 2.39]	0.003*
Legs fat mass (kg)	5.29 [3.44; 7.04]	4.27 [3.03; 6.23]	0.100
Android fat mass (kg)	2.12 [1.55; 2.77]	2.55 [1.47; 3.38]	0.143
Gynoid fat mass (kg)	3.21 [2.43; 4.10]	2.89 [2.11; 3.78]	0.122
Android to gynoid fat ratio	0.67 [0.56; 0.77]	0.83 [0.71; 0.93]	< 0.001**
Lumbar BMD (g/cm^2^)	1.05 [0.90; 1.17]	1.18 [1.06; 1.30]	0.001*
Left femoral neck BMD (g/cm^2^)	0.86 [0.76; 0.94]	0.94 [0.87; 1.06]	< 0.001**
Right femoral neck BMD (g/cm^2^)	0.88 [0.77; 0.92]	0.96 [0.88; 1.07]	< 0.001**
**Skinfold measurement**			
Chest SF (mm)	5.0 [4.0; 6.4]	5.5 [4.5; 7.0]	0.113
Tricep SF (mm)	20.5 [16.5; 23.9]	14 [11; 22.3]	0.004*
Midaxillary SF (mm)	19.1 [15.8; 24.0]	16.8 [13.0; 22.0]	0.105
Subscapular SF (mm)	19.3 [15.5; 22.9]	18.0 [16.0; 23.0]	0.920
Suprailiac SF (mm)	19.8 [16.1; 24.0]	17.0 [13.5; 21.8]	0.044*
Abdominal SF (mm)	24.0 [19.5; 28.3]	24.0 [18.0; 29.5]	0.725
Android SF (mm)	43.0 [35.1; 53.0]	41.0 [29.0; 52.5]	0.422
Thigh SF (mm)	19.3 [13.6; 25.9]	13.0 [9.0; 17.0]	0.001*
Thigh to android SF ratio	0.45 [0.34; 0.56]	0.32 [0.26; 0.38]	<0.001**
**Osteopenia % (n)**	40% (24)	44.2% (19)	0.671
**Osteoporosis % (n)**	28.3% (17)	16.3% (7)	0.154

Continuous variables were analyzed using the Mann-Whitney U-test and are presented as median values and [quartiles]; Categorical variables were analyzed using the Chi-square test and are presented as percentages (number). Abbreviations: DXA, dual-energy x-ray absorptiometry; BMC, bone mineral content; BMD, bone mineral density; SF, skinfold. Android skinfold was referred as the sum of abdominal and suprailiac skinfold thickness. *p < 0.05; **p < 0.001

### Gender-specific correlations of osteoporosis with grip strength and various body components in patients with T2DM

In order to evaluate if the occurrence of osteoporosis will company with changes of human body composition or muscular function independent from BMI, we subsequently examine the correlations of osteoporosis with grip strength and various body components in a gender-specific manner with BMI adjustment (Table 3). As expectation, osteoporosis was negatively correlated with bone mass (total mineral content, lumbar BMD, and bilateral femora neck BMD) in both genders, no matter adjusted by BMI or not. Of note, grip strength presents a significantly negative correlation with osteoporosis in both males and females, which maintains significance after BMI adjustment. Whereas there are clearly gender-specific differences regarding to the correlations of osteoporosis with fat mass and lean mass. In males, osteoporosis was negatively correlated with total and regional (arms, legs, android and gynoid) lean mass independently from BMI and osteoporosis was negatively correlated with total, regional (gynoid) fat mass and regional (midaxillary, subscapular and thigh) SF thickness specifically in females. The negative correlations between osteoporosis and regional fat mass or SF thickness in females are getting even stronger after BMI adjustment, particularly the lower-body fat (gynoid fat mass and thigh SF thickness). Female gender and age are well known risk factors for osteoporosis [3] and we can indeed observe a positive correlation between age and osteoporosis in females after adjusted by BMI, but this is non-significant in males.

**Table 3:** The gender-specific correlations of osteoporosis with grip strength and various body components in T2DM patients (60 females and 43 males)

Spearman correlation	Osteoporosis	
(*r*, *r* adjusted for BMI)	Female	Male
BMI	(-0.067; N/A)	(-0.193; N/A)
Age	(0.217; 0.259*)	(0.213; 0.235)
WHR	(-0.054; -0.058)	(-0.086; 0.154)
Grip strength	(-0.325*; -0.324*)	(-0.457**; -0.474**)
Total lean mass	(-0.089; -0.005)	(-0.579**; -0.485**)
Total fat mass	(-0.255*; -0.429**)	(-0.203; 0.052)
Total BMC	(-0.658**; -0.660**)	(-0.497**; -0.497**)
Arms lean mass	(-0.028; 0.074)	(-0.477**; -0.454**)
Legs lean mass	(-0.195; -0.135)	(-0.579**; -0.572**)
Android lean mass	(-0.031; 0.138)	(-0.340*; -0.321*)
Gynoid lean mass	(-0.230; -0.177)	(-0.485**; -0.559**)
Arms fat mass	(-0.193; -0.145)	(-0.168; 0.043)
Legs fat mass	(-0.250; -0.283*)	(-0.142; 0.088)
Android fat mass	(-0.224; -0.294*)	(-0.168; 0.066)
Gynoid fat mass	(-0.288*; -0.369**)	(-0.157; 0.073)
Lumbar BMD	(-0.714**; -0.630**)	(-0.508**; -0.476**)
Left femoral neck BMD	(-0.546**; -0.559**)	(-0.646**; -0.633**)
Right femoral neck BMD	(-0.554**; -0.556**)	(-0.606**; -0.671**)
Chest SF (mm)	(-0.151; -0.148)	(-0.031; -0.135)
Tricep SF (mm)	(-0.223; -0.225)	(-0.099; 0.046)
Midaxillary SF (mm)	(-0.294*; -0.319*)	(-0.170; -0.052)
Subscapular SF (mm)	(-0.262*; -0.301*)	(-0.198; -0.127)
Suprailiac SF (mm)	(-0.165; -0.157)	(-0.094; 0.093)
Abdominal SF (mm)	(-0.167; -0.172)	(-0.066; 0.131)
Thigh SF (mm)	(-0.370**; -0.391**)	(-0.058; 0.131)

Data were analyzed with Spearman correlation and presented with correlation coefficients (*r*; *r* adjusted for BMI). Abbreviation: BMI, body mass index; WHR, waist to hip ratio; BMC, bone mineral content; BMD, bone mineral density; SF, skinfold. *p < 0.05; **p < 0.01

### Correlations of thigh skinfold and grip strength with BMD among DM and non-DM individuals

For clarifying the potential utilization of thigh skinfold and grip strength in BMD assessment among DM and non-DM individuals, we further analyzed their correlations with lumbar/femoral neck T-score and osteoporosis in T2DM patients (60 females and 43 males), 45 non-DM females (age between 20 and 80 years) and 11 non-DM females (age between 50 and 80) as presented in Supplementary Tables 3 & 4. Intriguingly, only T2DM females showed a positive correlation between thigh SF and lumber T-score, which represented a negative correlation between thigh SF and osteoporosis even after adjustment for age and BMI. This phenomenon was not observed in non-DM females and T2DM males. Whereas grip strength showed positive correlations with lumbar or femoral neck T-score in all groups (T2DM females, T2DM males and non-DM females), which contributes to a negative correlation between grip strength and osteoporosis, and it was consistently maintained in T2DM patients even after age and BMI adjustment. Besides, we also evaluate the impact of DM duration and glycemic control on thigh skinfold and grip strength as shown in Supplementary Tables 5&6. Generally, both genders showed a trend of decreased grip strength and increased thigh SF thickness with longer DM duration (more than 10 years), but only female grip strength reached a significant difference. However, this is no significant difference in grip strength and thigh SF thickness between subjects with HbA1c less than 7% and more than 7%.

### Gender-specific difference of grip strength and various body components between osteoporotic and non-osteoporotic individuals with T2DM

In Table 4, we further separate the osteoporotic and non-osteoporotic patients with T2DM to compare their differences on grip strength and various body components with gender-specific manner. In line with the findings in Table 3, osteoporotic females have significantly lower grip strength with decreased gynoid fat mass and thinned SF on midaxillary, subscapular and thigh regions as compared to the non-osteoporotic ones. In turns of the males, the osteoporotic patients with T2DM present significant lower grip strength and decreased total and regional (arms, legs, android and gynoid) lean mass comparing to the non-osteoporotic ones.

**Table 4:** Gender-specific difference of grip strength and various body components between osteoporotic and non-osteoporotic T2DM patients

Female	Osteoporosis (n = 17)	Non-osteoporosis (n = 43)	*p* value
Age (years)	71.2 [65.9; 75.0]	68.7 [61.5; 71.8]	0.096
BMI (kg/m^2^)	24.5 [21.9; 26.9]	25.4 [21.8; 27.2]	0.605
Grip strength (kg)	15.8 [12.6; 17.9]	19.2 [15.8; 22.8]	0.013*
Total lean mass (kg)	31.9 [30.0; 39.2]	34.9 [33.1; 38.1]	0.496
Total fat mass (kg)	16.9 [13.0; 24.4]	22.8 [16.0; 25.7]	0.05
Arms lean mass (kg)	3.37 [2.93; 3.82]	3.44 [3.21; 3.93]	0.831
Legs lean mass (kg)	9.8 [8.7; 11.5]	10.5 [10.0; 11.2]	0.133
Android lean mass (kg)	2.38 [2.07; 3.26]	2.49 [2.16; 2.68]	0.812
Gynoid lean mass (kg)	4.31 [3.77; 4.92]	4.74 [4.32; 5.06]	0.078
Arms fat mass (kg)	1.95 [1.30; 2.64]	2.32 [1.72; 2.80]	0.138
Legs fat mass (kg)	4.38 [3.02; 6.00]	5.93 [3.72; 7.45]	0.055
Android fat mass (kg)	1.84 [1.40; 2.40]	2.34 [1.64; 2.87]	0.085
Gynoid fat mass (kg)	2.97 [2.16; 3.64]	3.43 [2.61; 4.28]	0.027*
Midaxillary SF (mm)	16.0 [13.3; 21.5]	21.0 [16.3; 25.0]	0.024*
Subscapular SF (mm)	15.5 [11.5; 22.0]	19.8 [17.0; 23.0]	0.044*
Thigh SF (mm)	13.8 [8.8; 20.0]	21.3 [15.0; 28.5]	0.005**
**Male**	**Osteoporosis (n = 7)**	**Non-osteoporosis (n = 36)**	***p*** **value**
Age (years)	69.0 [63.8; 74.6]	67.3 [59.4; 70.0]	0.176
BMI (kg/m^2^)	23.1 [19.7; 30.1]	26.3 [24.1; 29.7]	0.223
Grip strength (kg)	26.7 [14.2; 28.8]	37.1 [29.8; 39.8]	0.002**
Total lean mass (kg)	41.8 [41.2; 45.1]	51.7 [48.5; 55.4]	<0.001***
Total fat mass (kg)	11.3 [8.9; 31.5]	21.3 [13.4; 27.5]	0.198
Arms lean mass (kg)	4.61 [4.03; 5.06]	5.66 [5.11; 6.16]	0.001**
Legs lean mass (kg)	12.8 [11.8; 13.6]	15.7 [14.9; 17.0]	< 0.001***
Android lean mass (kg)	3.19 [2.71; 3.85]	3.74 [3.47; 4.13]	0.026*
Gynoid lean mass (kg)	5.44 [5.30; 6.61]	7.12 [6.63; 7.79]	0.001**
Arms fat mass (kg)	1.25 [0.83; 2.46]	1.56 [1.15; 2.35]	0.292
Legs fat mass (kg)	3.17 [2.33; 6.63]	4.28 [3.24; 6.18]	0.374
Android fat mass (kg)	1.44 [0.86; 3.85]	2.60 [1.64; 3.36]	0.292
Gynoid fat mass (kg)	2.09 [1.71; 4.22]	2.95 [2.18; 3.76]	0.323
Midaxillary SF (mm)	13.0 [8.0; 22.0]	16.9 [14.5; 22.5]	0.277
Subscapular SF (mm)	12.0 [11.0; 24.0]	18.5 [16.1; 23.0]	0.21
Thigh SF (mm)	11.0 [9.5; 18.3]	13.3 [8.8; 16.9]	0.711
**Female**	**Osteoporosis (n = 17)**	**Non-osteoporosis (n = 43)**	***p*** **value**
Age (years)	71.2 [65.9; 75.0]	68.7 [61.5; 71.8]	0.096
BMI (kg/m^2^)	24.5 [21.9; 26.9]	25.4 [21.8; 27.2]	0.605
Grip strength (kg)	15.8 [12.6; 17.9]	19.2 [15.8; 22.8]	0.013*
Total lean mass (kg)	31.9 [30.0; 39.2]	34.9 [33.1; 38.1]	0.496
Total fat mass (kg)	16.9 [13.0; 24.4]	22.8 [16.0; 25.7]	0.05
Arms lean mass (kg)	3.37 [2.93; 3.82]	3.44 [3.21; 3.93]	0.831
Legs lean mass (kg)	9.8 [8.7; 11.5]	10.5 [10.0; 11.2]	0.133
Android lean mass (kg)	2.38 [2.07; 3.26]	2.49 [2.16; 2.68]	0.812
Gynoid lean mass (kg)	4.31 [3.77; 4.92]	4.74 [4.32; 5.06]	0.078
Arms fat mass (kg)	1.95 [1.30; 2.64]	2.32 [1.72; 2.80]	0.138
Legs fat mass (kg)	4.38 [3.02; 6.00]	5.93 [3.72; 7.45]	0.055
Android fat mass (kg)	1.84 [1.40; 2.40]	2.34 [1.64; 2.87]	0.085
Gynoid fat mass (kg)	2.97 [2.16; 3.64]	3.43 [2.61; 4.28]	0.027*
Midaxillary SF (mm)	16.0 [13.3; 21.5]	21.0 [16.3; 25.0]	0.024*
Subscapular SF (mm)	15.5 [11.5; 22.0]	19.8 [17.0; 23.0]	0.044*
Thigh SF (mm)	13.8 [8.8; 20.0]	21.3 [15.0; 28.5]	0.005**
**Male**	**Osteoporosis (n = 7)**	**Non-osteoporosis (n = 36)**	***p*** **value**
Age (years)	69.0 [63.8; 74.6]	67.3 [59.4; 70.0]	0.176
BMI (kg/m^2^)	23.1 [19.7; 30.1]	26.3 [24.1; 29.7]	0.223
Grip strength (kg)	26.7 [14.2; 28.8]	37.1 [29.8; 39.8]	0.002**
Total lean mass (kg)	41.8 [41.2; 45.1]	51.7 [48.5; 55.4]	<0.001***
Total fat mass (kg)	11.3 [8.9; 31.5]	21.3 [13.4; 27.5]	0.198
Arms lean mass (kg)	4.61 [4.03; 5.06]	5.66 [5.11; 6.16]	0.001**
Legs lean mass (kg)	12.8 [11.8; 13.6]	15.7 [14.9; 17.0]	< 0.001***
Android lean mass (kg)	3.19 [2.71; 3.85]	3.74 [3.47; 4.13]	0.026*
Gynoid lean mass (kg)	5.44 [5.30; 6.61]	7.12 [6.63; 7.79]	0.001**
Arms fat mass (kg)	1.25 [0.83; 2.46]	1.56 [1.15; 2.35]	0.292
Legs fat mass (kg)	3.17 [2.33; 6.63]	4.28 [3.24; 6.18]	0.374
Android fat mass (kg)	1.44 [0.86; 3.85]	2.60 [1.64; 3.36]	0.292
Gynoid fat mass (kg)	2.09 [1.71; 4.22]	2.95 [2.18; 3.76]	0.323
Midaxillary SF (mm)	13.0 [8.0; 22.0]	16.9 [14.5; 22.5]	0.277
Subscapular SF (mm)	12.0 [11.0; 24.0]	18.5 [16.1; 23.0]	0.21
Thigh SF (mm)	11.0 [9.5; 18.3]	13.3 [8.8; 16.9]	0.711

Variables were analyzed using the Mann-Whitney U-test and are presented as median values and [quartiles]. Abbreviations: BMI, body mass index; SF, skinfold. *p < 0.05; **p < 0.01, ***p < 0.001

### Analyze gender-specific predictors for osteoporosis in patients with T2DM

Considering grip strength and SF thickness measurements could be easily performed in the outpatient department and might be practical tools for assessing the osteoporosis risk in patients with T2DM. We further evaluated if grip strength or regional SF thickness could work as predictors for the likelihood of being osteoporotic in patients with T2DM aged between 50 and 80 years. Variables including age, BMI, grip strength, smoking, alcohol drinking, and the SF parameters (subscapular SF, midaxillary SF and thigh SF) that presented significant difference between osteoporotic and non-osteoporotic individuals were selected for running the multivariable stepwise logistic regression in gender-specific way as shown in the Table 5. Our results disclosed that, in females, subjects with higher thigh SF have lower risk for osteoporosis (odds ratio: 0.889, *p* = 0.013) indicating every 1 mm increase in thigh SF thickness corresponded to a 11.1% decreased in the likelihood of having osteoporosis. Also, females with increased grip strength exist decreased risk for osteoporosis (odds ratio: 0.838, *p* = 0.026) indicating every 1 kg increase in grip strength corresponded to a 16.2% reduction in the likelihood of being osteoporotic. In males, SF parameters have no roles for predicting the risk of osteoporosis, whereas subjects with higher grip strength present lower risk for osteoporosis (odds ratio: 0.850, *p* = 0.008) indicating every 1 kg increase in grip strength corresponded a 15% reduction in the likelihood of having osteoporosis.

**Table 5:** Analyze gender-specific predictors for osteoporosis in T2DM patients

Female	Multivariable stepwise logistic regression	
	OR 95%CI *p-value*	
Thigh SF	**0.889 0.810–0.975 0.013**	
Grip strength	**0.838 0.718–0.979 0.026**	
**Male**	Multivariable stepwise logistic regression	
	OR 95%CI *p-value*	
Grip strength	**0.850 0.754–0.959 0.008**	

Variables including age, BMI, grip strength, subscapular SF, midaxillary SF, thigh SF, smoking, alcohol drinking were analyzed using multivariable stepwise logistic regression (Forward LR). Variables in females are entered in order as below: thigh SF, grip strength. Abbreviations: BMI, body mass index; SF, skinfold. Abbreviation: OR, odds ratio; CI, confidence interval

**Fig. 1:**
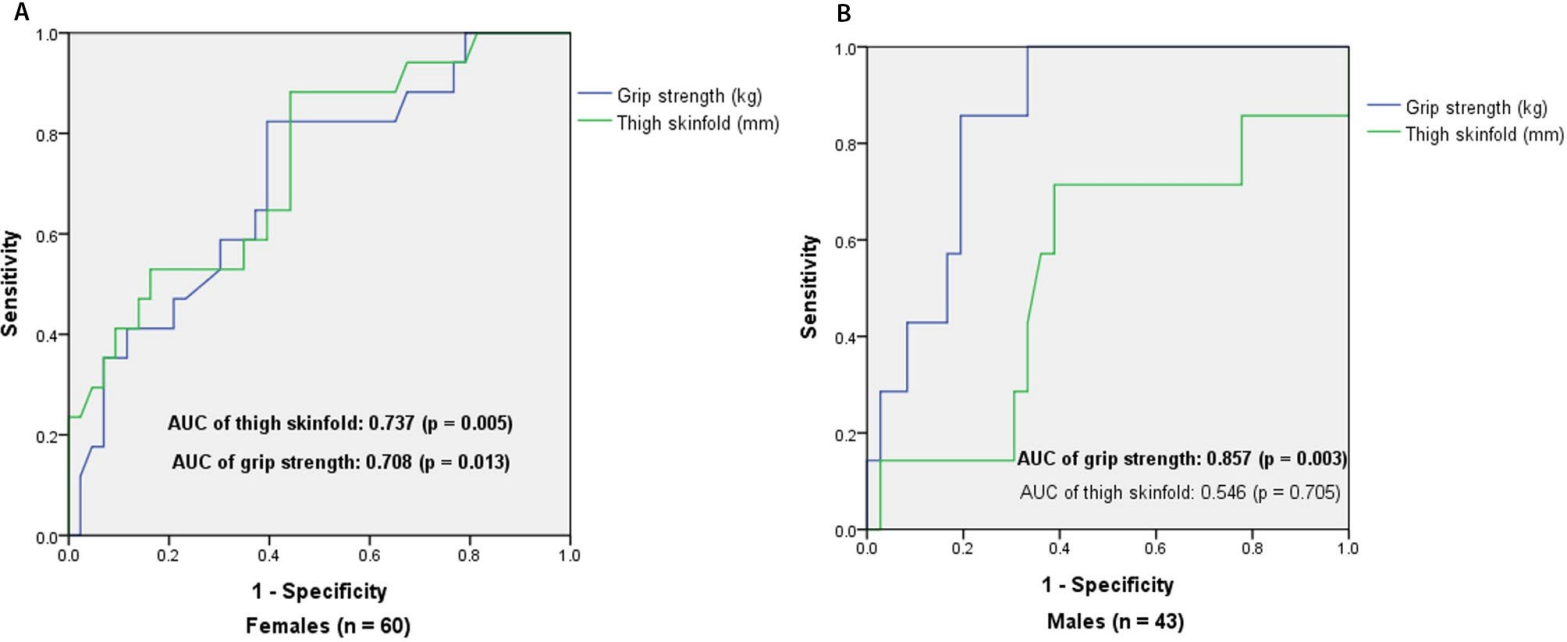
Receiver Operating Characteristic (ROC) curve of thigh skinfold and grip strength for predicting osteoporosis in T2DM patients ***(A)*** In females, cutoff levels of thigh skinfold (20.5 mm with sensitivity: 0.882 and specificity: 0.558) and grip strength (18.1 kg with sensitivity: 0.824 and specificity: 0.605) were identified. ***(B)*** In males, a cutoff levels of grip strength (29.0 kg with sensitivity: 0.857 and specificity: 0.806) was identified. The sum and individual value of sensitivity and specificity were used to determine the cutoff level. Abbreviation: AUC, area under the curve

Furthermore, ROC curve analysis was performed to assess how accurately the grip strength and thigh SF can distinguish T2DM patients with and without osteoporosis (Fig. 1). In females, grip strength and thigh SF both significantly distinguish these two groups with area under curve (AUC): 0.708 (*p* = 0.013) and 0.737 (*p* = 0.005), respectively. In males, only grip strength can significantly separate T2DM patients with osteoporosis from ones without osteoporosis with AUC: 0.857 (*p* = 0.003). In addition, the sum and individual values of sensitivity and specificity based on the ROC curve were used to determine gender-specific cutoff levels of grip strength and thigh SF for predicting osteoporosis. Our results disclosed 20.5 mm female thigh skinfold thickness (sensitivity: 0.882 and specificity: 0.558), 18.1 kg female grip strength (sensitivity: 0.824 and specificity: 0.605) and 29.0 kg male grip strength (sensitivity: 0.857 and specificity: 0.806) as reasonable cutoff levels for predicting osteoporosis in the Taiwanese patients with T2DM aged between 50 and 80 years.

## Discussion

In this study, we recruited Taiwanese patients with T2DM aged between 50 and 80 years to perform concurrent measurements of bone mineral density, body composition, skinfold thickness and dominant hand maximal grip strength. Our results revealed the osteopenia prevalence is more than 40% (40% in females and 44.2% in males) and the osteoporosis prevalence is 28.3% in females and 16.3% in males. Overall, more than 60% (68.3% in females and 60.5% in males) of patients with T2DM have concurrent metabolic disorders of bone tissues characterized by low bone mineral density (Table 2). Besides, we also disclosed there are BMI independent and gender-specific correlations of osteoporosis with body composition and grip strength. In both genders, grip strength was negatively correlated with osteoporosis. In males, lean mass presented a negative correlation with the occurrence of osteoporosis, whereas female lower-body fat (particular the gynoid fat mass and thigh SF thickness) was negatively correlated with the development of osteoporosis (Table 3). We further highlighted thigh SF thickness and grip strength might serve as a handy tool to predict the osteoporosis risk in patients with T2DM (Table 5; Fig. 1).

Overall, our results point out the importance to pay more attention to the risk of bone fragility in patients with T2DM. Previous studies have shown hyperglycemia involved in complicated dysregulation of bone metabolism. The BMD in T2DM patient could be normal or high compared to the matched controls without diabetes [27, 28], but the risk of fragility fracture is almost universally increased in patients with diabetes, particularly who have long duration of diabetes and poor glycemic control [29, 30]. Therefore, patients with T2DM have a paradoxically increased fracture risk even under relatively higher BMD. One possible explanation is patients with T2DM generally have higher BMI which will increase the weight loading effect to maintain or increase the BMD [31]. Also, T2DM related hyperglycemia and accumulation of advanced glycation end-products will decrease the overall bone turnover rate and cause micro and macro-architecture alternation to impair bone quality [32]. Moreover, diabetes associated retinopathy, polyneuropathy and occasional hypoglycemic episodes will further boost the risk of falls. Thus, detection of osteoporosis in patients with T2DM is a crucial issue for preventing the detrimental events of fragility fractures. Our data indicated the potential usefulness via applying thigh SF and grip strength measurements to identify the patients with T2DM with high risk for osteoporosis and will be easily conducted in the outpatient regular follow-up.

Studies to assess the impact of type 2 diabetes on the occurrence of osteoporosis showed inconsistent findings. Gudrun et al. [33] used DXA measured T-score on lumbar spine and femoral neck to identify osteoporosis and found patients with T2DM have lower prevalence rate than control groups, whereas Lin et al. [8] indicated Taiwanese patients with T2DM have greater risk of osteoporosis than ones without diabetes via applying a composite diagnosis of osteoporosis through ICD9-CM codes for osteoporosis, osteoporotic fractures, or usage of anti-osteoporotic agents. A recent meta-analysis including studies using DXA in China also supported patients with T2DM have a higher prevalence of osteoporosis [34]. These contradictory results might be due to different races, different methods for osteoporosis diagnosis (DXA or composited diagnosis codes), and different body sites (lumbar or hip or femoral neck) of DXA measurements since femoral neck tends to have lower BMD than lumbar spine in patients with T2DM [33]. In our study, we found the osteoporosis prevalence of patients with T2DM was 28.3% in females and 16.3% in males, which is lower than the pooled prevalence rate (44.8% in females and 37% in males) shown in the meta-analysis of China. In the meta-analysis, the authors further performed subgroup analysis and found there is higher osteoporosis prevalence in less developed regions and in the publication year before 2010 [34]. Since our data were collected between 2019 and 2020 in the Capital city of Taiwan, it is reasonable to observe a relatively decreased osteoporosis prevalence among both genders of patients with T2DM but will still require further larger database to demonstrate it.

Type 2 diabetes not only influences the bone metabolism, but also associated with compromised skeletal muscle mass [35] and muscular function [36]. Several mechanisms have linked the glucose dysmetabolism to lean mass dysfunction or loss. For example, insulin-activated pathways are involved in both glucose metabolism and protein synthesis of skeletal muscle. Also, diabetes associated obesity will increase the intramuscular lipid infiltration and lead to impairment of muscular function [37]. Notably, hand grip strength is a useful indicator for muscular function and weaker grip strength has been shown as an independent factor associated with increased falling and high mortality after hip fracture [38]. To the best of our knowledge, there is no research to examine the gender-specific associations of osteoporosis with body composition and hand grip strength in patients with T2DM. Our results disclosed loss of total and regional lean mass strongly correlated with increase of osteoporosis risk, which was solely observed in males and is independent from BMI. While the dominant hand grip strength presented a significantly and BMI-independently negative correlation with osteoporosis in both genders (Tables 3 and 4). We further highlighted grip strength measurement as a useful method to evaluate the likelihood of having osteoporosis in patients with T2DM (Table 5; Fig. 1). These results are in line with a recent investigation that pointed out hand grip strength was associated with bone microarchitecture and density and may serve as an easy assessment tool for identifying Asian men and women with a high risk of osteoporosis [39]. Our data further expands its potential utilization to the patients with T2DM.

Regarding to the association between lean mass and osteoporosis, previous reports showed conflicted findings. In older Chinese adults, InBody measured appendicular skeletal mass divided by the square of height was not associated with quantitative ultrasound defined osteoporosis at calcaneus [40]. Then, DXA defined osteoporosis was associated with low muscle mass measured by InBody equipment in elderly Korean women [41]. Whereas, in HIV-infected Indian men, low skeletal mass was associated with higher osteoporosis risk via using DXA analysis [42]. In this study, we applied DXA to concurrently define osteoporosis and quantify body composition in patients with T2DM and found there is a male gender-specific negative correlation between lean mass and osteoporosis (Table 3). Thus, our finding might be specific for patients with T2DM, but could also relate to different methods for measuring BMD and body composition. Further investigation will be still required to confirm our observation.

The potential roles of fat mass and body fat distribution on BMD or osteoporosis risk are just starting to be investigated in recent years. Using NHANES database to examine individuals under 60 years old, Rajesh et al. [43] found fat mass had a negative association with BMD, predominantly in men with high levels of fat. Whereas, in the elderly women with osteoporosis, fat mass presented a positive correlation with total body BMD [44]. When taking the fat distribution into consideration, central fat accumulation (increased android and visceral fat in men and increased visceral fat in women) were associated with lower BMD among 4,900 healthy individuals aged 30–50 years old from Oxford BioBank [45]. Likewise, android fat and visceral fat presented significant inverse associations with bone quality assessed by trabecular bone score in healthy Chinese men [46]. In patients with type 2 diabetes, as far as we know, there is only one study to investigate the association between fat distribution and the risk of osteoporosis. Yang et al. [47] performed cross-sectional analysis on 1,259 patients with T2DM aged 50 years or older and revealed higher leg fat mass was significantly correlated with lower osteoporosis risk among both genders with stronger effects in females. This report is compatible with our finding that gynoid fat, independent from BMI, was negatively correlated with the risk of osteoporosis, specifically in females (Table 3). Besides, we also applied non-DM females as the comparison and revealed the negative correlation between thigh SF and osteoporosis seems to be specific in T2DM females (Table 3 and Supplementary Table 3). Therefore, thigh SF thickness might serve as a useful predictor for assessing the risk of osteoporosis in female patients with T2DM (Table 5; Fig. 1). According to previous studies, reduction of regional SF thickness was associated with low bone density [48] or increased risk for hip fracture [49] and has been hypothesized that there are common pathophysiologic processes involved on osteoporotic bone and atrophic skin since both of them are composed more than 70% type I collagen [50]. Moreover, conjugated estrogen, as a well-known protective hormone for osteoporosis, was found to increase SF thickness at right great trochanter in postmenopausal women [51]. Therefore, our observation that thigh SF as a predictor for the risk of osteoporosis might represent the status of estrogen deficiency in female T2DM subjects and will need further research to support this theory.

Our study still has some limitations. First, the recruited participants are from the out-patient department of a single hospital with fair ambulatory activity, which limits the generalizability of our results. Second, we identified osteoporosis only based on the DXA measured T-score and individuals who met the clinical diagnosis of osteoporosis (osteopenia with history of fragility fracture) were not specifically selected. Therefore, the prevalence rate of osteoporosis observed in this study might be underestimated. Lastly, this is an observational cross-sectional study with relatively small sample sizes. Therefore, the clinical utilization of grip strength and thigh SF measurements for detection of osteoporosis in patients with T2DM will require further validation using a prospective multicenter cohort study in large population.

In conclusion, our results emphasized there is more than 60% prevalence rate of low BMD (osteopenia and osteoporosis) in patients with T2DM aged 50 years or older. We further revealed there are differentially gender-specific associations between osteoporosis and body composition. Particularly, our data suggested the measurement of hand grip strength and thigh SF thickness might be a practical tool for detection of osteoporosis in patients with T2DM.

## Electronic supplementary material

Below is the link to the electronic supplementary material.

Supplementary Material 1

## Data Availability

The data of this study are available from the corresponding author under reasonable request.
