# Supplementary material for: Gender-specific impacts of thigh skinfold thickness and grip strength for predicting osteoporosis in type 2 diabetes
Source: Diabetol Metab Syndr. 2023 May 18;15:103. doi: 10.1186/s13098-023-01087-w (PMC10193705; doi:10.1186/s13098-023-01087-w)
Supplement: Supplementary file 1 — Supplementary Material 1 [file 13098_2023_1087_MOESM1_ESM.docx]

**Supplementary Table 1. Basic anthropometric characteristics and biochemical data in the T2DM and non-DM females**

|  | T2DM females  Age 50-80 (n = 60) | Non-DM females  Age 20-80 (n = 45) | Non-DM females  Age 50-80 (n = 11) |
| --- | --- | --- | --- |
| Age (years)  BMI (kg/m^2^) | 68.8 [63.2; 72.6]  25.0 [21.9; 27.2] | **38.0 [29.2; 48.1]****  **22.0 [19.9; 23.5]**** | **65.4 [55.7; 68.4]***  22.2 [21.5; 25.1] |
| Waist-to-hip ratio | 0.94 [0.89; 1.01] | **0.82 [0.77; 0.91]**** | 0.93 [0.88; 0.98] |
| Systolic BP (mmHg) | 134 [123; 146] | **113 [108; 125]**** | 130 [113; 143] |
| Diastolic BP (mmHg) | 74 [67; 81] | **70 [64; 76]*** | 75 [64; 78] |
| Fasting glucose (mg/dL) | 129 [105; 153] | **89 [86; 97]**** | **101 [92; 113]*** |
| HbA1c % | 7.5 [6.6; 8.2] | **5.5 [5.2; 5.8]**** | **6.1 [5.8; 6.3]**** |
| LDL cholesterol (mg/dL) | 91 [71; 105] | **117 [92; 142]**** | **121 [98; 145]*** |
| Triglyceride (mg/dL) | 108 [77; 136] | **82 [55; 128]*** | **144 [119; 180]*** |
| Creatinine (mg/dL) | 0.7 [0.6; 0.8] | **0.7 [0.6; 0.7]*** | 0.7 [0.6; 0.7] |
| ALT (U/L) | 18 [15; 25] | **12 [10; 17]**** | 17 [13; 22] |
| Smoking % (n)  Alcohol drinking % (n) | 0 % (0)  1.7 % (1) | 2.2 % (1)  8.9 % (4) | 0 % (0)  0 % (0) |

Continuous variables were analyzed using the Mann-Whitney U-test and are presented as median values and [quartiles]; Categorical variables were analyzed using the Chi-square test and are presented as percentages (number). Abbreviations: BMI, body mass index; BP, blood pressure; HbA1c, glycated hemoglobin; LDL, low density lipoprotein; ALT, alanine aminotransferase. **p*<0.05; ***p*<0.001 as compared with T2DM females. The bold word refers to variables with *p*<0.05.

**Supplementary Table 2. Difference among grip strength, DXA scan parameters, skinfold measurement and osteoporosis/osteopenia in T2DM and non-DM females**

|  | T2DM females  Age 50-80 (n = 60) | Non-DM females  Age 20-80 (n = 45) | Non-DM females  Age 50-80 (n = 11) |
| --- | --- | --- | --- |
| **Grip strength (kg)** | 17.9 [15.2; 21.7] | **24.0 [20.3; 27.8]**** | **21.5 [18.7; 25.8]*** |
| **DXA scan parameters**  Total lean mass (kg)  Total fat mass (kg) | 34.7 [31.8; 38.3]  20.5 [15.3; 25] | 34.7 [32.3; 36.9]  **17.6 [13.7; 22.0]*** | 33.1 [29.4; 34.8]  19.7 [14.0; 21.9] |
| Total BMC (kg)  Arms lean mass (kg)  Legs lean mass (kg)  Android lean mass (kg)  Gynoid lean mass (kg)  Arms fat mass (kg)  Legs fat mass (kg)  Android fat mass (kg)  Gynoid fat mass (kg)  Android to gynoid fat ratio  Lumbar BMD (g/cm^2^)  Left femoral neck BMD (g/cm^2^)  Right femoral neck BMD (g/cm^2^)  **Skinfold measurement**  Chest SF (mm)  Tricep SF (mm)  Midaxillary SF (mm)  Subscapular SF (mm)  Suprailiac SF (mm)  Abdominal SF (mm)  Android SF (mm)  Thigh SF (mm)  Thigh to android SF ratio  **Osteopenia % (n)**  **Osteoporosis % (n)** | 1.92 [1.67; 2.21]  3.42 [3.07; 3.83]  10.4 [9.9; 11.2]  2.47 [2.15; 2.76]  4.68 [4.19; 5.01]  2.11 [1.59; 2.78]  5.29 [3.44; 7.04]  2.12 [1.55; 2.77]  3.21 [2.43; 4.10]  0.67 [0.56; 0.77]  1.05 [0.90; 1.17]  0.86 [0.76; 0.94]  0.88 [0.77; 0.92]  5.0 [4.0; 6.4]  20.5 [16.5; 23.9]  19.1 [15.8; 24.0]  19.3 [15.5; 22.9]  19.8 [16.1; 24.0]  24.0 [19.5; 28.3]  43.0 [35.1; 53.0]  19.3 [13.6; 25.9]  0.45 [0.34; 0.56]  40 % (24)  28.3 % (17) | **2.17 [1.94; 2.37]***  3.31 [3.00; 3.74]  **11.2 [10.2; 12.2]***  **2.20 [2.06; 2.39]***  4.88 [4.33; 5.37]  **1.55 [1.16; 2.06]****  **6.03 [5.00; 7.13]***  **1.42 [1.07; 2.08]****  **3.60 [3.18; 4.15]***  **0.39 [0.31; 0.49]****  **1.17 [1.06; 1.29]***  **0.94 [0.81; 1.02]***  **0.93 [0.81; 1.03]***  4.0 [3.8; 7.8]  18.0 [15.3; 20.6]  **15.0 [10.3; 19.3]***  **13.5 [11.9; 16.5]****  **16.0 [11.8; 18.0]****  **18.0 [15.0; 22.4]****  **33.8 [26.5; 40.0]****  20.0 [16.3; 25.6]  **0.59 [0.48; 0.78]****  31.1 % (14)  **8.9 % (4)*** | 1.86 [1.69; 2.17]  3.29 [2.87; 3.52]  10.3 [9.0; 11.1]  2.18 [2.00; 2.43]  4.45 [4.04; 4.61]  1.75 [1.47; 2.25]  5.88 [4.75; 7.15]  1.92 [1.42; 2.35]  3.54 [2.90; 3.77]  **0.56 [0.48; 0.68]***  0.97 [0.86; 1.17]  0.84 [0.73; 1.02]  0.86 [0.70; 0.99]  **4.0 [3.0; 4.75]***  18.0 [16.0; 26.0]  19.0 [15.5; 21.8]  **14.5 [12.8; 17.0]***  18.0 [13.3; 20.0]  19.0 [15.5; 26.0]  36.0 [26.8; 45.0]  20.0 [18.0; 24.5]  **0.59 [0.51; 0.65]***  36.4 % (4)  36.4 % (4) |

Continuous variables were analyzed using the Mann-Whitney U-test and are presented as median values and [quartiles]; Categorical variables were analyzed using the Chi-square test and are presented as percentages (number). Abbreviations: DXA, dual-energy x-ray absorptiometry; BMC, bone mineral content; BMD, bone mineral density; SF, skinfold. Android skinfold was referred as the sum of abdominal and suprailiac skinfold thickness. **p*<0.05; ***p*<0.001 as compared with T2DM females. The bold word refers to variables with *p*<0.05.

**Supplementary Table 3. The correlations of thigh skinfold with lumbar/femoral neck T-score, and osteoporosis in T2DM patients and non-DM females**

| Spearman correlation  (*r*, *r* adjusted for age & BMI) | Thigh SF | | | |  |
| --- | --- | --- | --- | --- | --- |
|  | T2DM female  Age 50-80  (n=60) | T2DM male  Age 50-80  (n=43) | Non-DM female  Age 20-80  (n=45) | Non-DM female  Age 50-80  (n=11) |  |
| BMI  Age  Lumbar T-score  Left femoral neck T-score  Right femoral neck T-score | (**0.561****; N/A)  (-0.162; N/A)  (**0.366****; 0.241)  (0.213; 0.117)  (0.215; 0.107) | (**0.608****; N/A)  (0.096; N/A)  (0.071; -0.033)  (0.018; -0.068)  (0.097; -0.103) | (0.054; N/A)  (0.108; N/A)  (-0.154; -0.143)  (-0.064; -0.110)  (-0.085; -0.108) | (0.236; N/A)  (0.155; N/A)  (-0.192; -0.091)  (0.173; 0.230)  (0.169; 0.260) |  |
| Osteoporosis | (**-0.370****; **-0.349****) | (-0.058; 0.102) | (0.066; 0.031) | (0.239; 0.188) |  |

Data were analyzed with Spearman correlation and presented with correlation coefficients (*r*; *r* adjusted for BMI). Abbreviation: BMI, body mass index; WHR, waist to hip ratio; BMC, bone mineral content; BMD, bone mineral density; SF, skinfold. **p*<0.05; ***p*<0.01. The bold word refers to variables with *p*<0.05.

**Supplementary Table 4. The correlations of grip strength with lumbar/femoral neck T-score, and osteoporosis in T2DM patients and non-DM females**

| Spearman correlation  (*r*, *r* adjusted for age & BMI) | Grip strength | | | |  |
| --- | --- | --- | --- | --- | --- |
|  | T2DM female  Age 50-80  (n=60) | T2DM male  Age 50-80  (n=43) | Non-DM female  Age 20-80  (n=45) | Non-DM female  Age 50-80  (n=11) |  |
| BMI  Age  Lumbar T-score  Left femoral neck T-score  Right femoral neck T-score | (0.129; N/A)  (-0.228; N/A)  (0.156; 0.032)  (**0.257***; 0.116)  (0.189; 0.042) | (0.002; N/A)  (**-0.425****; N/A)  (**0.481****; **0.378***)  (**0.541****; **0.493****)  (**0.500****; **0.483****) | (**0.329***; N/A)  (-0.028; N/A)  (**0.463****; **0.302***)  (**0.417****; 0.274)  (**0.408****; 0.220) | (0.118; N/A)  (**-0.636***; N/A)  (**0.744****; 0.321)  (0.564; 0.479)  (0.579; 0.305) |  |
| Osteoporosis | (**-0.325***; **-0.265***) | (**-0.457****; **-0.430****) | (**-0.385****; -0.240) | (**-0.657***; -0.408) |  |

Data were analyzed with Spearman correlation and presented with correlation coefficients (*r*; *r* adjusted for BMI). Abbreviation: BMI, body mass index; WHR, waist to hip ratio; BMC, bone mineral content; BMD, bone mineral density; SF, skinfold. **p*<0.05; ***p*<0.01. The bold word refers to variables with *p*<0.05.

**Supplementary Table 5. Difference of grip strength and thigh skinfold among individuals with DM duration less than 10 years and more than 10 years**

| **T2DM female** |  | DM duration<10  (n =27) | DM duration≥10  (n =33) | *p* value |
| --- | --- | --- | --- | --- |
| Grip strength (kg)  Thigh SF (mm) |  | 19.5 [16.1; 23.5]  17.8 [14.5; 25.8] | 16.7 [12.9; 20.7]  20.0 [11.5; 29.0] | **0.022***  0.876 |
| **T2DM male** |  | DM duration<10  (n =16) | DM duration≥10  (n =27) | *p* value |
| Grip strength (kg)  Thigh SF (mm) |  | 37.1 [27.4; 41.3]  11.5 [8.5; 16.5] | 31.7 [27.0; 38.1]  13.0 [10.0; 19.0] | 0.333  0.232 |

Variables were analyzed using the Mann-Whitney U-test and are presented as median values and [quartiles]. Abbreviation: SF, skinfold. **p*<0.05; ***p*<0.01, ****p*<0.001. The bold word refers to variables with *p*<0.05.

**Supplementary Table 6. Difference of grip strength and thigh skinfold among individuals with glycated hemoglobin less than 7 % and more than 7 %**

| **T2DM female** |  | HbA1c<7%  (n =20) | HbA1c≥7%  (n =40) | *p* value |
| --- | --- | --- | --- | --- |
| Grip strength (kg)  Thigh SF (mm) |  | 17.2 [15.4; 21.4]  17.4 [15.0; 24.5] | 18.1 [15.1; 22.7]  19.8 [13.1; 26.4] | 0.718  0.857 |
| **T2DM male** |  | HbA1c<7%  (n =17) | HbA1c≥7%  (n =26) | *p* value |
| Grip strength (kg)  Thigh SF (mm) |  | 37.4 [26.7; 44.7]  12.0 [8.5; 17.6] | 32.7 [27.3; 37.9]  13.0 [9.4; 17.1] | 0.303  0.610 |

Variables were analyzed using the Mann-Whitney U-test and are presented as median values and [quartiles]. Abbreviation: SF, skinfold. **p*<0.05; ***p*<0.01, ****p*<0.001.
